# Associations of zinc-α-2-glycoprotein with metabolic syndrome and its components among adult Arabs

**DOI:** 10.1038/s41598-022-09022-1

**Published:** 2022-03-22

**Authors:** Amal M. Alenad, Lamya F. Alkaltham, Shaun Sabico, Malak N. K. Khattak, Kaiser Wani, Nasser M. Al-Daghri, Majed S. Alokail

**Affiliations:** Chair for Biomarkers of Chronic Diseases, Biochemistry Department, College of Science, King Saud University, PO Box, 2455, Riyadh, 11451 Saudi Arabia

**Keywords:** Biochemistry, Biomarkers, Endocrinology

## Abstract

Epidemiological studies suggest that the Zinc-α-2-glycoprotein (ZAG) plays significant physiological roles. In this study we investigate whether ZAG could be considered as a clinical biomarker in the diagnosis and prognosis of metabolic syndrome (MetS) in Saudi population. As such insights urgently required for management of MetS. Thus, we have determined serum levels of ZAG in patients with MetS and normal individuals. We have also assessed the correlation between ZAG and different components of MetS. In this case–control study, clinical information of 200 Saudi male and female subjects (age range 30–65) with MetS (n = 100) and healthy controls (n = 100) were extracted from the database of the Chair of Biomarkers of Chronic Disease (CBCD) in King Saud University (KSU), Riyadh, Saudi Arabia. MetS was screened according to NCEP ATP III criteria (National Cholesterol Education Program Adult Treatment Panel III). Fasting glucose and lipid profile levels were measured using Konelab. Serum TNF-α, IL- 6, CRP and ZAG levels were measured using commercially available assays. There was an age-dependent significant increase in ZAG level among MetS subjects than controls (43.8 ± 19.5 vs 48.1 ± 14.8; *P* = 0.04). A significant inverse correlation between ZAG and serum HDL-cholesterol (r = − 0.20, *P* < 0.05) was observed. Whereas, triglycerides (r = 0.25, *P* < 0.01), waist circumference (WHR) (r = 0.17, *P* < 0.05) and CRP (r = 0.24, *P* < 0.01) were all significantly and positively associated with ZAG. Circulating ZAG is associated with MetS in an age-dependent manner. Serum ZAG is a potential biomarker for MetS.

## Introduction

Metabolic syndrome (MetS) is a complicated disorder. Progressions in proinflammatory and prothrombotic states are potential mechanisms contributing to the pathophysiology of MetS, due to adipocytokine production. There are also several established mechanisms in the etiology of MetS which involves the insulin resistance (IR) with fatty acid flux, low-level chronic inflammation and oxidative stress^1^. Obesity-mediated MetS and IR are highly prevalent in the Saudi society. In a study involving 17,293 Saudi patients, the prevalence of MetS was 39.3%^2^. Moreover, females have a higher MetS prevalence than males^2,3^. Low-level chronic inflammation combined with increased fat mass and damaging lipid profiles is associated with the pathophysiology of MetS and IR^1^. Increase in adipocyte has been shown to cause abundance of proinflammatory cytokines such as interleukin (IL)-6, resistin, tumor necrosis factor alpha (TNF-α) and C-reactive protein (CRP), and this could be due the presence of macrophages in adipose tissue which might be partially the source of the production of proinflammatory cytokines^4^. IR also was found to be associated with overproduction of proinflammatory cytokines and relative deficiency of adiponectin in muscle and liver^4^.

Growing evidence suggests that altered production of adipose-derived protein factors such as Zinc-α2-glycoprotein (ZAG) plays an important role in the pathophysiology of obesity and its associated complications such as MetS^5,6^. ZAG gene expression in adipocytes is primarily controlled by androgens and progestins, and Glucocorticoids^7^. Epidemiological studies showed that, the levels of serum ZAG were positively correlated with the levels of serum triglycerides (TAG) and adipocyte fatty acid-binding protein, whereas the levels of ZAG were inversely associated with HDL-cholesterol^8^. Overexpression of ZAG in cultured hepatocytes significantly inhibited lipogenesis by decrease metabolic nuclear receptors sterol regulatory element-binding protein (SREBP-1c), liver X receptor (LXR) and lipogenic enzymes in the liver, also lipolysis and fatty acid β-oxidation were stimulated. On the other hand, knocking down of ZAG resulted in inhibition of fatty acid β-oxidation, increased lipogenesis and lipid accumulation^9^. Findings of these studies suggest an important role of ZAG in lipolysis, and that the way ZAG is involved in lipid metabolism, is by enhancing the conversion of white adipose tissue into brown adipose tissue. The suggested mechanism of ZAG exerting its effect on white adipose tissue, is by increasing the expression of the peroxisome proliferator-activated receptor c (PPARc) and early B cell factor 2, that are considered in promoting cascade expression of genes involved in browning of the adipose tissue^10^.

We hypothesized that MetS components have different effects on the circulating levels of ZAG. Therefore, the aim of this study was to investigate the relationship between circulating ZAG level and MetS as well as its components, to provide an insight for predicting the occurrence of MetS in Saudi patients. Additional aim of the study was to assess circulating levels of ZAG and proinflammatory cytokines, CRP, IL- 6 and TNF-α in patients with or without MetS.

## Materials and method

### Subjects

A total of 200 adult Saudi subjects (94 men; 106 women), aged 23–65 years were randomly selected from the database of Biomarker Screening in Riyadh Project (RIYADH COHORT) collected by Center for Biomarkers in Chronic Diseases (CBCD) in Riyadh, KSA^11,12^. All participants provided written and informed consent prior to inclusion. Ethical approval was granted by the Ethics Committee of the College of Science Research Center, King Saud University, Riyadh, Kingdom of Saudi Arabia (KSA). Participants completed a questionnaire their general health status, demographic information, and past medical history. Anthropometric and biochemical data from the database was utilized to assess the status of full MetS and its five components as present/absent (dichotomous data) according to the criteria set in the National Cholesterol Education Programme Adult Treatment Panel III (NCEP-ATP III) where MetS was present when at least three out of the five following components are present: Waist circumference of > 88 cm; fasting glucose > 5.6 mmol/L; HDL-cholesterol < 1.30 mmol/L; triglycerides > 1.7 mmol/L; systolic blood pressure > 130 mmHg and/or diastolic blood pressure > 85 mmHg^13^.

The sample size was calculated based on an earlier study by Yeung et al.^8^ where serum Zinc-alpha2-Glycoprotein in MetS patients was reported with an effect size of > 0.5. Thus, a total sample size of 176 subjects (88 per group) was required to detect the effect size of 0.5 with 95% power using 5% significance level. Based on this, 100 MetS and 100 non-MetS subjects were selected from the database by random selection based on the RAND function in the Microsoft excel. Prior to the final selection, the subjects with reported chronic conditions like liver, kidney, heart failures and pregnant women were removed from the selection and the process was repeated to get the final count of 100 in each group.

### Inclusion and exclusion criteria

Subjects on anti-hyperglycemic treatment; pregnant or lactating women; with known renal, hepatic, pulmonary, cardiac, etc., complications were excluded from this study.

### Anthropometry and blood collection

Anthropometry included height (cm), weight (kg), waist and hip circumference (cm), and mean systolic and diastolic blood pressure (mmHg, average of two reading). Body mass index (BMI) was calculated as weight in kilograms divided by height in square meters. Waist-hip ratio (WHR) was calculated as the quotient between waist and hip circumferences. They were asked to fast 10 h or overnight before blood withdrawal. Fasting blood samples (> 10 h) were collected and transferred immediately to a non-heparinized tube for centrifugation. The collected serum was transferred to pre-labeled new tubes, kept on ice, and delivered to the CBCD in King Saud University, Riyadh, KSA, for immediate storage at − 20 °C.

### Blood chemistry

Fasting blood glucose and lipid profile (triglycerides, total and HDL-cholesterol) were assessed using routine chemical methods (Konelab analyzer, Espoo Finland). Hypertriglyceridemia was defined as circulating triglycerides ≥ 1.7 mmol/l was considered abnormal level^13^. Low HDL-cholesterol was defined as < 1.03 mmol/l and total cholesterol HDL ratio > 3.5. Low-HDL level for women was set at < 1.3 mmol/l^14,15^.

### Inflammatory markers and ZAG

Serum TNF- α and IL-6 level were determined using MILLIPLEX MAP human adipokine magnetic bead panel 2 kit obtained from Millipore Corporation. Serum human CRP level were determined using ELISA kits obtained from R&D System, USA. Serum ZAG level were determined using ELISA kits using BioVendor-Laboratory Medicine.

### Data analysis

Data were analyzed using SPSS (version 22 Chicago, IL, USA). Continuous data were presented as mean ± standard deviation (SD) for normal variables and non-Gaussian variables were presented in median (1st and 3rd) percentiles. Categorical data were presented as frequencies and percentages (%). All continuous variables were checked for normality using Kolmogorov–Smirnov test. Non-Gaussian variables were log-transformed prior to parametric analysis. Independent T-test and Mann Whitney U were used to compare mean differences in Gaussian and Non-Gaussian variables. Correlations between variables were done using Pearson’s and spearman correlation analysis. Stepwise regression analysis was performed for dependent predicators of ZAG (ug/ml) and area under the curve (AUC) was calculated using receiver operating characteristic (ROC) curve to determine viability of ZAG as a biomarker for MetS and its components. An AUC of 0.9 to 1 is considered excellent, 0.8 to 0.9 is considered good, 0.7 to 0.8 is considered fair, 0.6 to 0.7 is considered poor, and 0.5 to 0.6 is considered very poor. A p-value < 0.05 was considered statistically significant.

### Institutional review board statement

Ethical approval was obtained from the Ethics Committee of the College of Science Research Center, King Saud University, Riyadh, Saudi Arabia (approval No. E-20-5369). All methods were carried out in accordance with relevant guidelines and regulations.

### Informed consent statement

Written informed consent was obtained from all subjects involved in the study.

## Results

### General characteristics of the study subjects

A total of 200 subjects (94 males and 106 females), age range 30–65; mean BMI 29.8 ± 5.5 kg/m^2^) participated in this study. Half of all subjects had MetS, with significant increase in blood pressure, fasting glucose and triglycerides. Also, there was a decrease in HDL-cholesterol among the MetS subjects. TNF- α and CRP were significantly elevated in MetS subjects than normal subjects (Table 1).

**Table 1:** Clinical characteristics of all subjects.

Parameters	Normal	MetS	*P*-value	*P*-value*
N	100 (50/50)	100 (44/56)		
Age (years)	35.4 ± 7.9	41.9 ± 7.2	< 0.001	
Height (cm)	164.0 ± 10.1	164 .1 ± 9.9	0.99	0.54
Weight (kg)	74.7 ± 17.1	85.5 ± 14.3	< 0.001	< 0.001
BMI (kg/m^2^)	27.7 ± 5.2	31.9 ± 5.1	< 0.001	< 0.001
Waist (cm)	87.6 ± 14.9	105.6 ± 15.5	< 0.001	< 0.001
Hips (cm)	104.8 ± 12.9	108.5 ± 14.2	0.053	0.1
WHR	0.84 ± 0.11	0.98 ± 0.17	< 0.001	< 0.001
SBP (mm Hg)	117.4 ± 12.2	129.7 ± 17.5	< 0.001	< 0.001
DBP (mm Hg)	70.9 ± 9.2	78.9 ± 11.4	< 0.001	< 0.001
**Lipid Profiles**				
Total Cholesterol (mmo/l)	5.2 ± 1.0	5.5 ± 1.6	0.19	0.37
HDL-Cholesterol (mmol/l)	1.23 ± 0.3	1.03 ± 0.3	< 0.001	< 0.001
LDL-Cholesterol	3.4 ± 0.9	3.3 ± 1.4	0.78	0.53
Triglycerides (mmol/l) #	1.34 ± 0.7	2.37 ± 0.8	< 0.001	< 0.001
**Glycemic profile**				
Glucose (mmol/l)	5.2 ± 0.7	7.1 ± 3.3	< 0.001	< 0.001
**Inflammatory Markers**				
IL-6 (pg/ml)	2.6 (1.5–5.0)	1.9 (0.9–4.1)	0.2	0.23
TNF- α (pg/ml)	0.41 (0.1–1.5)	1.5 (0.8–1.8)	< 0.001	< 0.001
CRP (ng/ml)	2010 (578–4441)	4388 (1724–6185)	< 0.001	< 0.001
**Protein**				
ZAG (ug/ml)	43.8 ± 19.5	48.1 ± 14.8	0.08	0.04

Data presented in mean ± SD and median (25th–75th) percentiles.

*Denotes *P*-value adjusted for age *P*-value significant at *P* < 0.05, 0.01 level.

### Correlation of ZAG level with proinflammatory cytokines in different metabolic component

**Figure 1:**
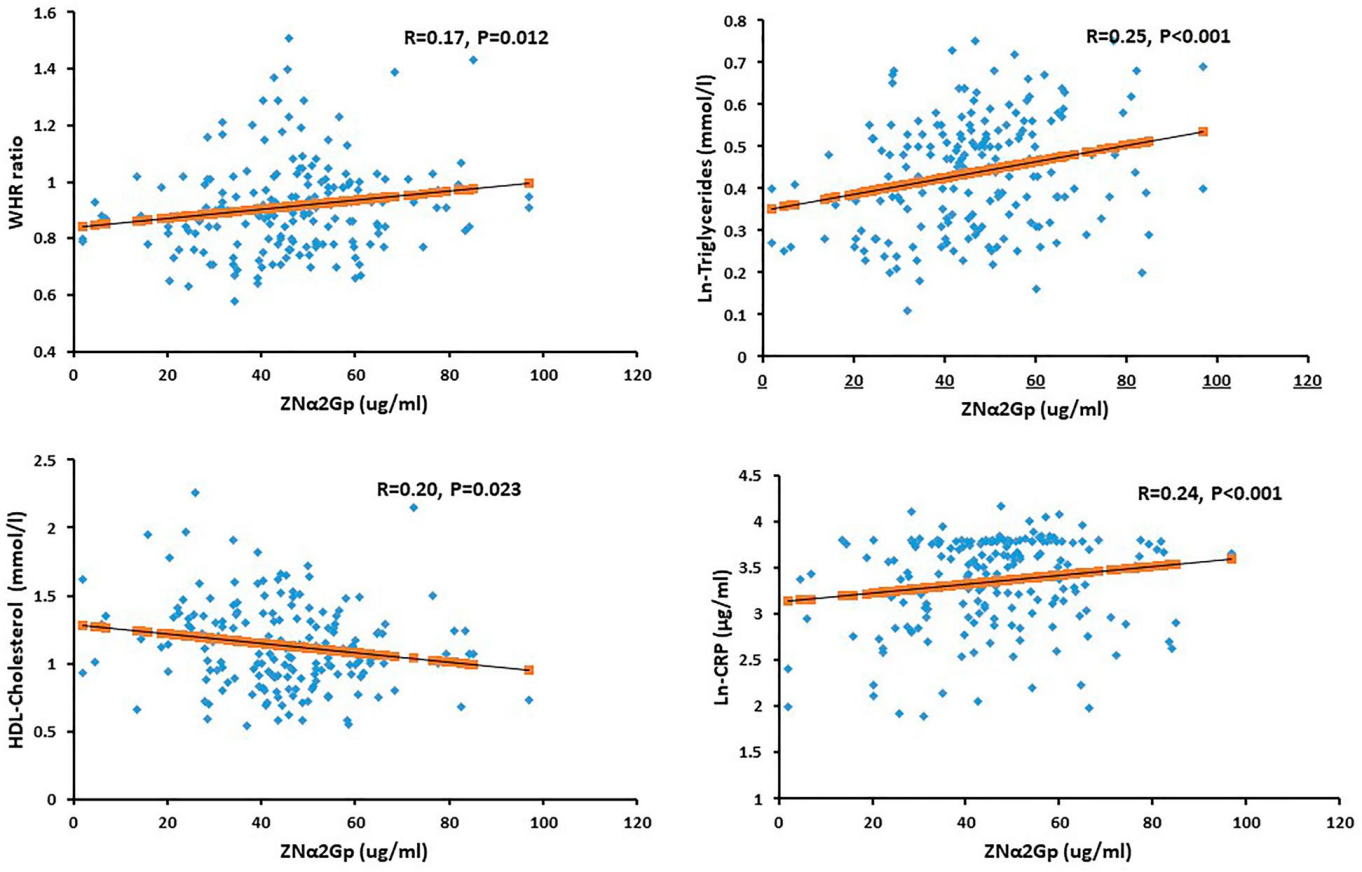
Bivariate associations of ZAG with Cardiometabolic Parameters.

A significant inverse correlation between ZAG and serum HDL-cholesterol (R = − 0.20, *P* < 0.05) was observed. Whereas, triglycerides (R = 0.25, *P* < 0.01), waist circumference (WHR) (R = 0.17, *P* < 0.05) and CRP (R = 0.24, *P* < 0.01) were all significantly and positively associated with ZAG (Fig. 1). We also observed a positive correlation between ZAG and CRP in non-central obesity group (R = 0.31, *P* < 0.01), as well in female group (R = 0.42, *P* < 0.01). Also, CRP and ZAG have positive correlations with the hypertensive group (R = 0.28, *P* < 0.05), and in hypertensive female group (R = 0.40, *P* < 0.01). While TNF-α and ZAG have an inverse correlation in non-hypertensive males (R = − 0.47, *P* < 0.05). Furthermore, there was an inverse correlation between IL-6 and ZAG level in the hyperglycemic group in all subjects (R = − 0.26, *P* < 0.05). In the female group with normal HDL-cholesterol and triglycerides level, there were significant positive correlations between CRP and ZAG (R = 0.44, *P* < 0.01), (R = 0.35, *P* < 0.05) respectively (Table 2).

**Table 2:** Correlation analysis between ZAG (ug/ml) with Proinflammatory Cytokines in association with different metabolic components.

Parameters	All		Males		Females	
	No	Yes	No	Yes	No	Yes
**Central Obesity: Waist circumference > 101.6 cm (males), > 88.9 cm (females)**						
IL-6 (pg/ml)	− 0.10	− 0.13	− 0.14	− 0.20	− 0.06	− 0.07
TNF- α (pg/ml)	0.00	− 0.06	0.07	− 0.23	0.00	0.05
CRP (µg/ml)	0.31**	− 0.01	0.21	− 0.12	0.42**	0.07
**Hypertension: Systolic BP > 130 mmHg and/or diastolic BP > 85 mmHg**						
IL-6 (pg/ml)	− 0.08	− 0.16	− 0.08	− 0.23	− 0.09	− 0.08
TNF- α (pg/ml)	− 0.09	− 0.02	− 0.47*	0.12	0.09	− 0.01
CRP (µg/ml)	0.11	0.28*	0.08	0.04	0.15	0.40**
**Hyperglycemia: > 5.6 mmol/l**						
IL-6 (pg/ml)	0.03	− 0.26*	− 0.06	− 0.27	0.09	− 0.25
TNF- α (pg/ml)	− 0.20	0.13	− 0.08	− 0.08	− 0.14	0.22
CRP (µg/ml)	0.09	0.27*	− 0.06	0.29	0.25	0.28
**Hypertriglyceridemia: ≥ 1.7 mmol/l**						
IL-6 (pg/ml)	− 0.13	− 0.05	− 0.19	− 0.05	− 0.09	− 0.05
TNF- α (pg/ml)	0.05	− 0.21	− 0.48	− 0.10	0.20	− 0.22
CRP (µg/ml)	0.15	0.12	− 0.13	0.14	0.35*	0.10
**Low HDL-Cholesterol: < 1.03 mmol/l (males), < 1.3 mmol/l (females)**						
IL-6 (pg/ml)	− 0.08	− 0.13	0.08	− 0.33	− 0.21	0.08
TNF- α (pg/ml)	− 0.11	− 0.02	− 0.55	0.16	0.18	− 0.09
CRP (µg/ml)	0.22	0.10	0.02	0.12	0.44**	0.10

Data presented as coefficient (R).

*Denotes significance at 0.05 level.

**Denotes significance at 0.01 level.

Logistic regression analysis was carried out to identify the relationship between MetS components and ZAG tertiles. The odds-ratios for hypertriglyceridemia were significantly higher for 2nd [OR: 3.7 (1.6–8.3); *P* = 0.001) and 3rd [OR: 2.9 (1.3–6.4); *P* = 0.009] tertiles than the first tertile in the multivariate model. Furthermore, the odds ratios of MetS were also significantly higher for 2nd [OR: 3.0 (1.3–6.6); *P* = 0.006] and 3rd [OR: 2.3 (1.1–5.1); *P* = 0.04] tertiles than the first tertile in the multivariate model. The rest of the odds ratios were not significant (Table 3).

**Table 3:** Logistic Regression analysis for ZAG tertile on MetS and its components.

Quartile	Crude		Multivariate Model	
	OR (95% CI)	*P*-Value	OR (95% CI)	*P*-Value
**Central obesity**				
1 (< 40.3)	1		1	
2 (40.4–51.7)	1.6 (0.8–3.1)	0.22	1.8 (0.8–3.6)	0.13
3 (> 51.7)	1.7 (0.9–3.5)	0.13	1.9 (0.9–3.9)	0.1
**Hypertension**				
1 (< 40.3)	1		1	
2 (40.4–51.7)	0.6 (0.3–1.3)	0.22	0.7 (0.3–1.4)	0.27
3 (> 51.7)	0.6 (0.3–1.2)	0.17	0.6 (0.3–1.3)	0.16
**Hyperglycemia**				
1 (< 40.3)	1		1	
2 (40.4–51.7)	1.0 (0.5–2.0)	0.98	1.1 (0.5–2.3)	0.79
3 (> 51.7)	1.0 (0.5–2.1)	0.94	1.0 (0.5–2.2)	0.89
**Hypertriglyceridemia**				
1 (< 40.3)	1		1	
2 (40.4–51.7)	2.8 (1.4–5.7)	0.005	3.7 (1.6–8.3)	0.001
3 (> 51.7)	2.4 (1.2–4.9)	0.02	2.9 (1.3–6.4)	0.009
**Low HDL-Cholesterol**				
1 (< 40.3)	1		1	
2 (40.4–51.7)	1.8 (0.8–3.6)	0.11	1.8 (0.8–3.7)	0.11
3 (> 51.7)	1.4 (0.7–2.9)	0.31	1.4 (0.7–2.9)	0.31
**MetS**				
1 (< 40.3)	1		1	
2 (40.4–51.7)	2.3 (1.1–4.6)	0.02	3.0 (1.3–6.6)	0.006
3 (> 51.7)	1.9 (0.9–4.0)	0.062	2.3 (1.1–5.1)	0.04

Data presented Odd Ratio (95% CI). *P*-value < 0.05, 0.01 level will be significant. Multivariate logistic regression analysis was adjusted for age.

**Figure 2:**
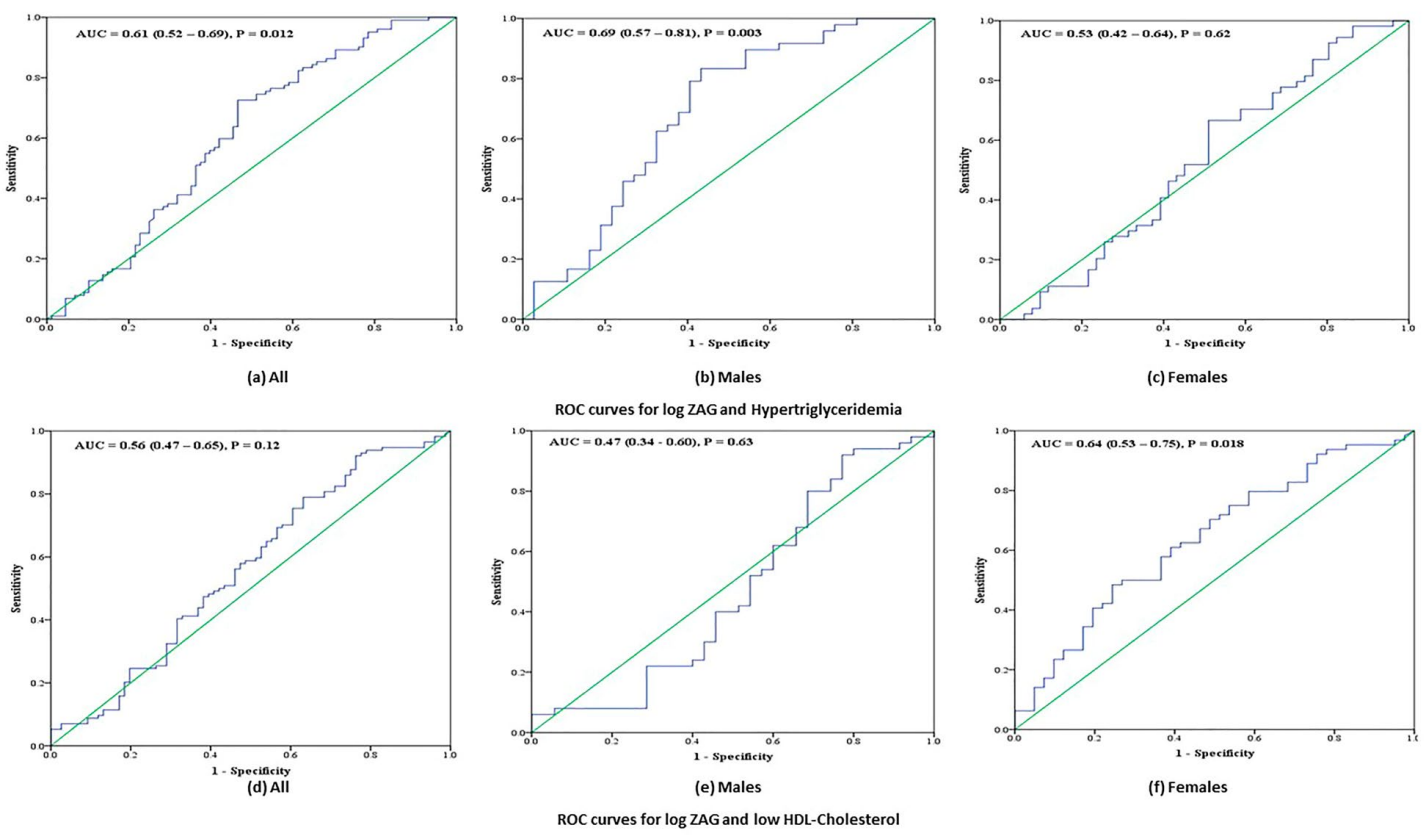
AUC for ZAG and MetS in a) All; b) Males; c) Females as well as AUC for ZAG and Hypertriglyceridemia in d) All; e) Males and f) Females.

Lastly, AUC was done to determine whether ZAG can be used as a clinical biomarker for the diagnosis of MetS and its components (Table 4). In all subjects, ZAG was not a sensitive biomarker for MetS [AUC 0.57 (95% CI 0.5–0.6; *P* = 0.10] and a significant but poor biomarker of hypertriglyeridemia [0.61 (0.5–0.7; *P* = 0.01)] with sensitivity and specificity of 59.8% and 58.0% respectively (ZAG cutoff: 45.5 ug/ml). When stratified according to sex, ZAG is a fair biomarker for hypertriglyceridemia in males [0.7 (95% CI 0.6–0.8; *P* = 0.003] with sensitivity and specificity of 68.8% and 62.2% respectively (ZAG cutoff: 44.9 ug/ml). Furthermore, ZAG is a poor but significant biomarker for low-HDL cholesterol in females [0.6 (95% CI 0.5–0.8; *P* = 0.02] with sensitivity and specificity of 60.9% and 61.0% respectively (ZAG cutoff: 44.3 ug/ml) (Fig. 2).

**Table 4:** Area under the curve for metabolic syndrome and its components against ZAG.

MetS and Components	All		Males		Females	
	AUC	*P*-value	AUC	*P*-value	AUC	*P*-value
MetS	0.57 (0.49–0.65)	0.10	0.54 (0.42–0.67)	0.515	0.59 (0.48–0.70)	0.11
Obesity	0.55 (0.47–0.64)	0.21	0.54 (0.42–0.66)	0.568	0.58 (0.46–0.68)	0.24
Hypertension	0.47 (0.38–0.55)	0.43	0.50 (0.37–0.63)	0.971	0.45 (0.33–0.56)	0.35
High Glucose	0.50 (0.42–0.58)	0.95	0.45 (0.33–0.58)	0.464	0.54 (0.42–0.65)	0.54
High Triglycerides	0.61 (0.52–0.69)	0.01	0.69 (0.57–0.81)	0.003	0.53 (0.42–0.64)	0.62
Low HDL	0.56 (0.47–0.65)	0.12	0.47 (0.34–0.60)	0.63	0.64 (0.53–0.75)	0.018

Data presented as Area Under the Curve (AUC) and 95% CI. *P*-value < 0.05 considered significant.

## Discussion

In this study we investigated the relationship between circulating ZAG concentrations and MetS components, and analyze the effects of the circulating levels of ZAG on proinflammatory cytokines, CRP and TNF-α in Saudi adults with and without MetS. To the best of our knowledge, there are a few studies on ZAG level in MetS patient worldwide, and this study is the first to be conducted in Saudi Arabia. The current study showed that circulating ZAG levels in MetS patients were significantly higher than in those without MetS in age dependent manner. In contrast, the results of a study by Lei et al.^16^ and Wang et al.^17^ revealed that circulating ZAG levels were lower in MetS subjects than in those without MetS. This controversy between the findings could be due to differences in the study design, including sample size, age, and more importantly subjects involved in the two studies were from different population. Age effect on ZAG levels was confirmed in a recent study by Tan et al.^18^ were they reported significantly lower circulating ZAG level in young women with MetS components. Whereas, in agreement with findings of our study, the results of Yeung et al.^8^ study on Chinese subjects have shown that ZAG levels were significantly elevated in MetS subjects and it was also elevated progressively with an increasing number of components of the MetS. Our results revealed that ZAG also correlate positively with some of MetS component and significantly with triglycerides, waist and inversely correlated with HDL-cholesterol. Similar findings were shown by previous study^8^. Animal studies have shown that ZAG act as a regulator of lipid metabolism, and our results multiplied these finding and we showed significantly positive correlation between ZAG and triglyceride, and inverse correlation with HDL-cholesterol^18,19^. These clinical finding suggest that ZAG may play an important regulatory role in lipid metabolism in humans as well. As ZAG have been shown to possess beneficial effect on mice obesity reduction, decreasing body fat content, and stimulating lipolysis in differentiated adipocytes in vitro^18^. Based on research finding we speculate that, the observed elevation of serum level of ZAG in MetS patient could be a compensatory process for the human body to overcome the metabolic stress induced by obesity. Alternatively, obesity, could be contributing to ZAG resistance which leads to its upregulation. SBP, DBP and Glucose level were significantly higher in MetS patient than the healthy control, but there was no significant correlation between ZAG and hypertension or fasting glucose. However, in previous studies, it was found that there is an inverse correlation between ZAG and hypertension^16^. Elevation of visceral adipocytes in MetS disturbs the adipocytokine secretion and leads to a low-grade chronic inflammatory state by the macrophages of adipocytes. This inflammatory state is found to be associated with IR and MetS^20^. CRP is well known marker for obesity-related inflammation and the Mets, thus we measured its levels. Subjects with MetS had markedly higher CRP level compared to patients without MetS, this finding came in agreement with other study^21^. CRP level also was significantly higher in obese patient than normal subjects. Furthermore, both CRP and ZAG have been found significantly and positively associated with each other^8^. Apparently, inflammatory cytokines are able to induce IR in both adipose tissue and muscle.in case of obesity, adipocytes produce plenty of cytokines such as TNF-α and IL-6, the primary stimulator of the production of CRP in the liver. These responses seem to link MetS and inflammation, and explain the increase of CRP level in MetS patients, and the strong relationship between BMI and CRP levels^8,22^.

Proinflammatory TNF-α plays a crucial role in the inflammatory cytokine cascade that is involved in multiple disease pathogenesis. TNF-α has been studied by Gormez et al.^21^ and it was found significantly higher in MetS patient than normal subjects. The significant increase in TNF-α level was due to the increased its gene expression in Mets patient compare to control^21^. In addition, TNF-α level was found to be higher in obese patients than their non-obese counterparts^23^. We found that TNF-α was higher in female than male. Interestingly, our data reveals a positive correlation between TNF-α and ZAG in normal subjects but inverse correlation in MetS subjects. This inverse correlation was reported in cultured human adipocytes upon treatment with TNF-α, which lowered ZAG mRNA levels. Among inflammatory markers, TNF-α is one of the most important inflammatory cytokines that is critically involved in the development of insulin resistance and pathogenesis of T2DM. Additionally, neutralization of TNF-α also improved the hepatic IR in animal model, also TNF-α-deficient mice have shown improved insulin sensitivity^24,25^. With regards to the proinflammatory cytokine IL-6 levels, there was no significant change in IL-6 found in both groups, but there was a significant decrease in male subjects with MetS as compared to normal male subjects. Also, there was an inverse correlation between IL-6 and ZAG in normal and MetS subjects. We have no knowledge of any previous study that studied the relationship between ZAG and IL-6. IL-6 was found to be elevated in obesity and type 2 diabetes mellitus patients, while Circulating ZAG levels in the serum and adipose tissue of obese patients and obese mice are notably lower in compare to normal subjects and ZAG were found to be oppositely associated with insulin resistance in type 2 diabetes mellites patients^26–29^.

## Conclusions

This study shows that circulating ZAG levels in patients with MetS are elevated compared to healthy controls. ZAG was positively associated with MetS component like triglycerides, waist as well with inflammatory CRP, while inversely correlated with HDL-cholesterol. These findings support the pathological role of ZAG in human obesity and its related metabolic disorders. Further studies involving bigger size populations are needed to validate the use of ZAG as a biomarker for MetS and other cardiometabolic disorders.

## Supplementary Information

Supplementary Information.

## Data Availability

All data are available within the article.
