# Supplementary material for: Associations of zinc-α-2-glycoprotein with metabolic syndrome and its components among adult Arabs
Source: Sci Rep. 2022 Mar 22;12:4908. doi: 10.1038/s41598-022-09022-1 (PMC8941079; doi:10.1038/s41598-022-09022-1)
Supplement: Supplementary file 1 — Supplementary Information. [file 41598_2022_9022_MOESM1_ESM.docx]

**Supplementary Table S1. Clinical Characteristics of Metabolic Components in All patients**

| **Parameters** | **All** | | |
| --- | --- | --- | --- |
| **Central Obesity: Waist circumference >101.6 cm (males), >88.9 cm (females)** | | | |
|  | **Waist<** | **Waist>** | **P-value** |
| N | 94 | 106 |  |
| IL-6 (pg/ml) | 2.1(1.1-4.5) | 2.4(1.3-4.4) | 0.88 |
| TNF- α (pg/ml) | 0.6(0.3-1.5) | 1.2(0.7-1.8) | **0.003** |
| CRP (µg/ml) | 2.1(0.7-4.5) | 4.3(1.3-6.2) | **<0.001** |
| ZAG (ug/ml) | 44.6±18.2 | 47.3±16.5 | 0.28 |
| **Hypertension: Systolic blood pressure >130 mmHg and/or diastolic blood pressure >85 mmHg** | | | |
|  | <130/85 | >130/85 |  |
| N | 122 | 78 |  |
| IL-6 (pg/ml) | 2.2(0.9-4.1) | 2.6(1.5-4.6) | 0.44 |
| TNF- α (pg/ml) | 0.8(0.4-1.5) | 1.3(0.8-1.8) | **0.01** |
| CRP (µg/ml) | 2.7(0.8-5.4) | 4.2(1.5-6.2) | **0.01** |
| ZAG (ug/ml) | 46.6±17.2 | 45.2±17.6 | 0.57 |
| **Hyperglycemia: Fasting glucose >5.6 mmol/l** | | | |
| N | 112 (<=5.6) | 88 (>5.6) |  |
| IL-6 (pg/ml) | 2.03(1.1-4.7) | 2.6(1.1-4.1) | 0.62 |
| TNF- α (pg/ml) | 1.04(0.4-1.6) | 1.1(0.5-1.7) | 0.23 |
| CRP (µg/ml) | 2.7(0.8-5.8) | 3.8(1.3-6.1) | 0.2 |
| ZAG (ug/ml) | 45.9±18.1 | 46.1±16.5 | 0.959 |
| **Hypertriglyceridemia: Serum triglycerides ≥1.7 mmol/l** | | | |
| N | 97 (<1.7) | 103 (≥1.7) |  |
| IL-6 (pg/ml) | 2.7(1.5-5.2) | 2.0 (0.9-4.0) | 0.14 |
| TNF- α (pg/ml) | 0.62(0.2-1.2) | 1.4(0.8-1.8) | **<0.001** |
| CRP (µg/ml) | 2.7(0.7-5.9) | 3.8(1.5-6.0) | 0.05 |
| ZAG (ug/ml) | 42.6±19.5 | 48.9±14.6 | **0.01** |
| **Low HDL-Cholesterol: Serum HDL-cholesterol <1.03 mmol/l (males), <1.30 mmol/l (females)** | | | |
| N | 83 (>1.03,1.3) | 117 (<1.03,1.3) |  |
| IL-6 (pg/ml) | 2.6(1.4-6.7) | 2.1(1.1-3.5) | 0.16 |
| TNF- α (pg/ml) | 0.7(0.2-1.6) | 1.1(0.6-1.6) | **0.03** |
| CRP (µg/ml) | 2.7(0.8-4.9) | 3.9(1.3-6.2) | 0.06 |
| ZAG (ug/ml) | 43.8±18.1 | 47.6±16.7 | 0.14 |

Note: Data presented in mean±SD and median (25^th^ -75^th^) percentiles. P-value significant at p<0.05, 0.01 level

**Supplementary Table S2. Clinical Characteristics of Metabolic Components in Males**

| **Parameters** | **Males** | | |
| --- | --- | --- | --- |
| **Central Obesity: Waist circumference >101.6 cm (males)** | | | |
|  | **Waist< 101.6 cm** | **Waist>101.6** | **P-value** |
| N | 48 | 46 |  |
| IL-6 (pg/ml) | 2.2(1.1-3.7) | 2.6(1.1-4.6) | 0.77 |
| TNF- α (pg/ml) | 0.6(0.2-1.1) | 1.04(0.6-1.6) | 0.06 |
| CRP (µg/ml) | 1.9(0.9-4.2) | 4.4(1.6-6.2) | **0.004** |
| ZAG (ug/ml) | 46.9±20.5 | 47.7±17.3 | 0.87 |
| **Hypertension: Systolic blood pressure >130 mmHg and/or diastolic blood pressure >85 mmHg** | | | |
|  | <130/85 | >130/85 |  |
| N | 61 | 33 |  |
| IL-6 (pg/ml) | 2.6(1.4-6.8) | 2.3(0.8-4.6) | 0.79 |
| TNF- α (pg/ml) | 0.8(0.2-1.5) | 1.0 (0.6-1.5) | 0.26 |
| CRP (µg/ml) | 2.4(0.9-5.2) | 3.8(1.8-5.9) | 0.07 |
| ZAG (ug/ml) | 47.3±19.1 | 47.4±18.5 | 0.99 |
| **Hyperglycemia: Fasting glucose >5.6 mmol/l** | | | |
| N | 53 (<=5.6) | 41 (>5.6) |  |
| IL-6 (pg/ml) | 2.7(1.6-4.4) | 2.0 (0.8-3.8) | 0.32 |
| TNF- α (pg/ml) | 0.6(0.2-1.1) | 1.3(0.8-1.6) | **0.008** |
| CRP (µg/ml) | 1.9(0.8-4.1) | 4.6(2.0-6.2) | **0.003** |
| ZAG (ug/ml) | 48.1±20.2 | 46.5±17.2 | 0.69 |
| **Hypertriglyceridemia: Serum triglycerides ≥1.7 mmol/l** | | | |
| N | 45 (<1.7) | 49 (≥1.7) |  |
| IL-6 (pg/ml) | 3.3(1.5-5.3) | 1.9(0.8-3.2) | 0.11 |
| TNF- α (pg/ml) | 0.21(0.1-0.9) | 1.1(0.8-1.6) | **0.002** |
| CRP (µg/ml) | 2.5(0.8-5.6) | 3.5(1.7-5.7) | 0.11 |
| ZAG (ug/ml) | 40.4±20.6 | 52.7±15.4 | **0.002** |
| **Low HDL-Cholesterol: Serum HDL-cholesterol <1.03 mmol/l (males)** | | | |
| N | 42 (>1.3) | 52(<1.3) |  |
| IL-6 (pg/ml) | 3.4(1.9-5.9) | 1.7(0.8-3.2) | **0.02** |
| TNF- α (pg/ml) | 0.2(0.1-1.4) | 1.1(0.7-1.6) | **0.02** |
| CRP (µg/ml) | 2.7(0.9-4.6) | 3.5(1.6-5.9) | 0.14 |
| ZAG (ug/ml) | 47.6±20.5 | 47.1±17.2 | 0.9 |

Note: Data presented in mean±SD and median (25^th^ -75^th^) percentiles. P-value significant at p<0.05, 0.01 level

**Supplementary Table S3. Clinical Characteristics of Metabolic Components in Female Patients**

| **Parameters** | **Females** | | |
| --- | --- | --- | --- |
| **Central Obesity: Waist circumference >101.6 cm (males), >88.9 cm (females)** | | | |
|  | **Waist<** | **Waist>88.9** | **P-value** |
| N | 46 | 60 |  |
| IL-6 (pg/ml) | 2.03(1.1-5.0) | 2.2 (1.4-4.1) | 0.69 |
| TNF- α (pg/ml) | 0.7(0.4-1.5) | 1.3(0.7-1.8) | **0.02** |
| CRP (µg/ml) | 2.4(0.7-5.8) | 4.3(1.3-6.2) | **0.04** |
| ZAG (ug/ml) | 42.4±15.8 | 46.9±16.1 | 0.15 |
| **Hypertension: Systolic blood pressure >130 mmHg and/or diastolic blood pressure >85 mmHg** | | | |
|  | <130/85 | >130/85 |  |
| N | 61 | 45 |  |
| IL-6 (pg/ml) | 1.9(0.9-4.9) | 2.6(1.7-3.7) | 0.15 |
| TNF- α (pg/ml) | 0.96(0.4-1.6) | 1.5(0.8-1.9) | **0.02** |
| CRP (µg/ml) | 2.8(0.7-5.9) | 4.7(1.3-6.2) | 0.099 |
| ZAG (ug/ml) | 45.9±15.4 | 43.7±16.9 | 0.46 |
| **Hyperglycemia: Fasting glucose >5.6 mmol/l** | | | |
| N | 59 (<=5.6) | 47 (>5.6) |  |
| IL-6 (pg/ml) | 1.9(0.9-4.7) | 2.9(1.4-5.0) | 0.11 |
| TNF- α (pg/ml) | 1.2(0.6-1.8) | 1.1(0.5-1.8) | 0.69 |
| CRP (µg/ml) | 4.2(0.8-6.2) | 2.8(1.1-6.0) | 0.36 |
| ZAG (ug/ml) | 44.3±16.1 | 45.8±16.0 | 0.64 |
| **Hypertriglyceridemia: Serum triglycerides ≥1.7 mmol/l** | | | |
| N | 52 (<1.7) | 54(≥1.7) |  |
| IL-6 (pg/ml) | 2.1(1.1-4.9) | 2.0(1.4-4.7) | 0.56 |
| TNF- α (pg/ml) | 0.8(0.4-1.4) | 1.5(0.9-2.1) | **0.001** |
| CRP (µg/ml) | 3.2(0.6-6.1) | 4.1(1.3-6.2) | 0.21 |
| ZAG (ug/ml) | 44.3±18.8 | 45.7±13.1 | 0.65 |
| **Low HDL-Cholesterol: Serum HDL-cholesterol <1.03 mmol/l (males), <1.30 mmol/l (females)** | | | |
| N | 41 (>1.03) | 65 (<1.03) |  |
| IL-6 (pg/ml) | 1.9(0.9-8.6) | 2.3(1.4-3.5) | 0.86 |
| TNF- α (pg/ml) | 1.0(0.4-1.7) | 1.2(0.6-1.8) | 0.27 |
| CRP (µg/ml) | 2.9(0.7-5.6) | 4.2(1.0-6.2) | 0.21 |
| ZAG (ug/ml) | 40.5±15.3 | 47.9±15.9 | **0.02** |

Note: Data presented in mean±SD and median (25^th^ -75^th^) percentiles. P-value significant at p<0.05, 0.01 level

**Supplementary Table S4: Correlation of ZNα2Gp (ug/ml) with other parameters**

| Parameters | All | Gender | | | | | | MetS Status | |
| --- | --- | --- | --- | --- | --- | --- | --- | --- | --- |
|  |  | Males | | | Females | | | Normal | MetS |
|  |  | All | Normal | MetS | All | Normal | MetS |  |  |
| N | 200 | 94 | 50 | 44 | 106 | 44 | 56 | 100 (50/50) | 100 (44/56) |
| Age (years) | -0.02 | 0.04 | 0.01 | 0.03 | -0.10 | -0.20 | -0.12 | -0.10 | -0.05 |
| Height (cm) | 0.10 | 0.15 | **0.31*** | -0.19 | -0.13 | -0.14 | -0.10 | 0.20 | -0.10 |
| Weight (kg) | **0.15*** | 0.13 | 0.27 | -0.17 | 0.11 | -0.11 | 0.22 | 0.14 | 0.10 |
| BMI (kg/m2) | 0.12 | 0.14 | 0.19 | -0.01 | 0.14 | -0.15 | **0.30*** | 0.04 | 0.16 |
| Waists | **0.15*** | 0.03 | 0.18 | **-0.31*** | **0.24*** | 0.07 | 0.26 | 0.17 | 0.01 |
| Hip | 0.02 | 0.05 | 0.23 | -0.27 | -0.01 | -0.12 | 0.07 | 0.10 | -0.10 |
| WHR | **0.17*** | 0.10 | 0.14 | -0.10 | **0.22*** | 0.18 | 0.16 | 0.17 | 0.10 |
| SBP (mm Hg) | -0.06 | -0.10 | -0.16 | -0.10 | -0.04 | **-0.32*** | -0.03 | -0.21 | -0.05 |
| DBP (mm Hg) | -0.05 | 0.02 | 0.03 | -0.10 | -0.10 | -0.24 | -0.10 | -0.11 | -0.10 |
| Glucose (mmol/l) | 0.06 | 0.02 | 0.05 | -0.09 | 0.10 | -0.13 | 0.13 | -0.05 | 0.03 |
| Cholesterol (mmo/l) | 0.05 | 0.16 | 0.18 | 0.16 | -0.05 | 0.05 | -0.16 | 0.11 | -0.02 |
| HDL-Cholesterol (mmol/l) | **-0.20*** | -0.15 | -0.21 | 0.14 | **-0.22*** | **-0.34*** | -0.10 | **-0.26*** | -0.03 |
| Triglycerides (mmol/l)# | **0.25**** | **0.40**** | **0.46**** | **0.36*** | 0.10 | 0.06 | -0.14 | **0.30**** | 0.10 |
| TNF- Alpha (pg/ml)# | 0.05 | -0.19 | -0.17 | 0.06 | 0.15 | 0.11 | -0.16 | 0.10 | -0.10 |
| IL-1Beta (pg/ml)# | **0.17*** | 0.16 | 0.24 | 0.01 | 0.19 | **0.35*** | -0.19 | **0.31**** | -0.10 |
| CRP (µg/ml)# | **0.24**** | 0.06 | 0.06 | -0.02 | **0.36**** | **0.34*** | 0.10 | **0.27*** | 0.04 |
| IL-18 (pg/ml) | 0.07 | -0.03 | -0.04 | 0.01 | 0.18 | 0.22 | **0.37**** | -0.06 | **0.24*** |
| Insulin (µIU/ml) | 0.12 | -0.02 | 0.13 | -0.16 | **0.23*** | 0.10 | 0.26 | 0.18 | 0.01 |

Note: Data presented as coefficient (R); * denotes significance at 0.05 level; ** denotes significance at 0.01 level.
